# Supplementary material for: Stability of peatland carbon to rising temperatures
Source: Nat Commun. 2016 Dec 13;7:13723. doi: 10.1038/ncomms13723 (PMC5159855; doi:10.1038/ncomms13723)
Supplement: Supplementary Information — Supplementary Figures 1-12, Supplementary Table 1 and Supplementary References [file ncomms13723-s1.pdf]

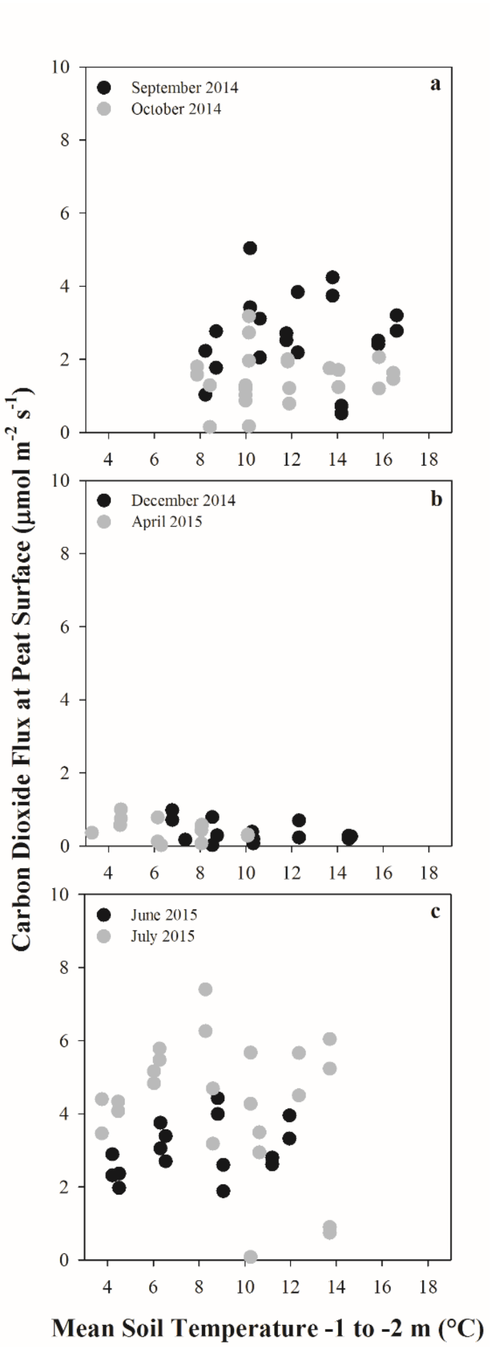

Supplementary Figure 1: Seasonal CO_2 flux from the S1 bog

The seasonal CO_2 flux from 1.2 m diameter collars during (a) fall 2014, (b) winter 2015, (c) and summer 2015 across temperature treatments. Black and gray dots distinguish between daily averages for two different sampling points during the season. No significant correlations between CO_2 flux and temperature were observed during these measurement periods.

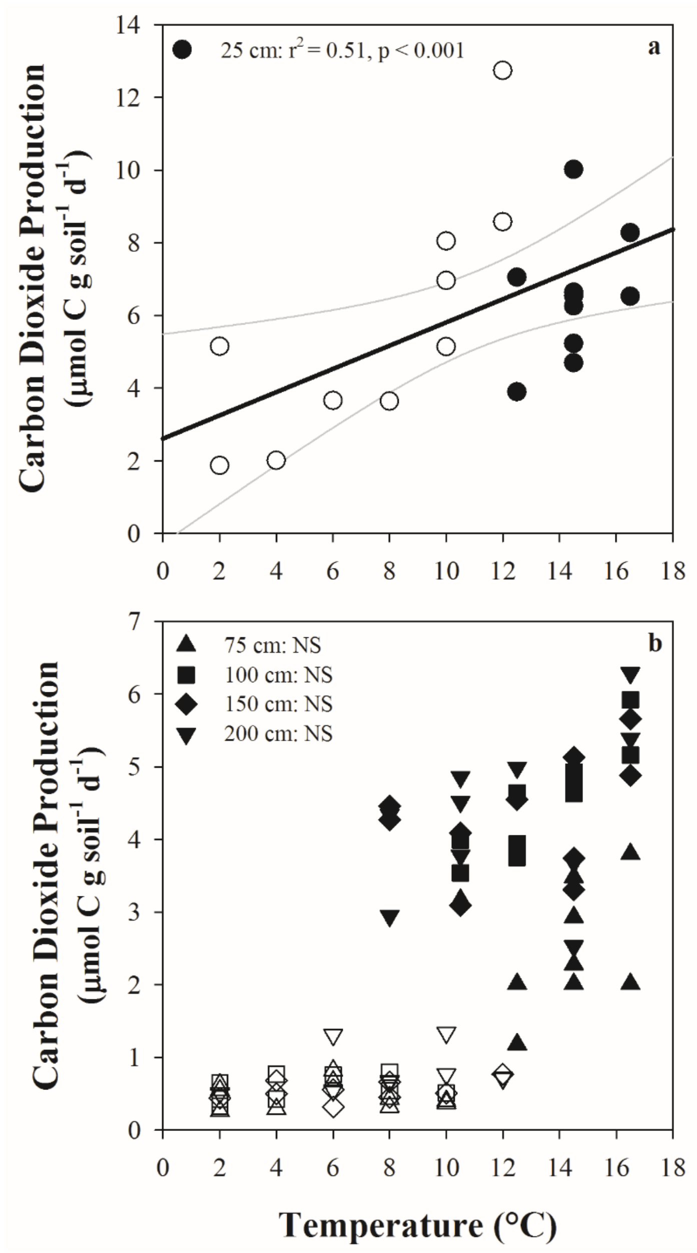

Supplementary Figure 2: CO_2 production in peat incubations

The temperature response of CO_2 production observed from peat samples taken from (a) 25 cm and (b) at depth (75 – 200 cm). Anaerobic incubations were completed within 1ºC of in situ temperatures after approximately 4 (closed symbols, September 2014) and 13 (open symbols, June 2015) months of DPH. The circles indicate results from peat collected from 25 cm, triangles indicate results from 75 cm, squares indicate results from 100 cm, diamonds indicate results from 150 cm, and inverted triangles represent results from 200 cm. The temperature response at depth was analyzed by season due to a distinct bimodal distribution. The black line in panel (a) indicates the linear regression line with grey lines denoting the 95% confidence interval. NS = not significant.

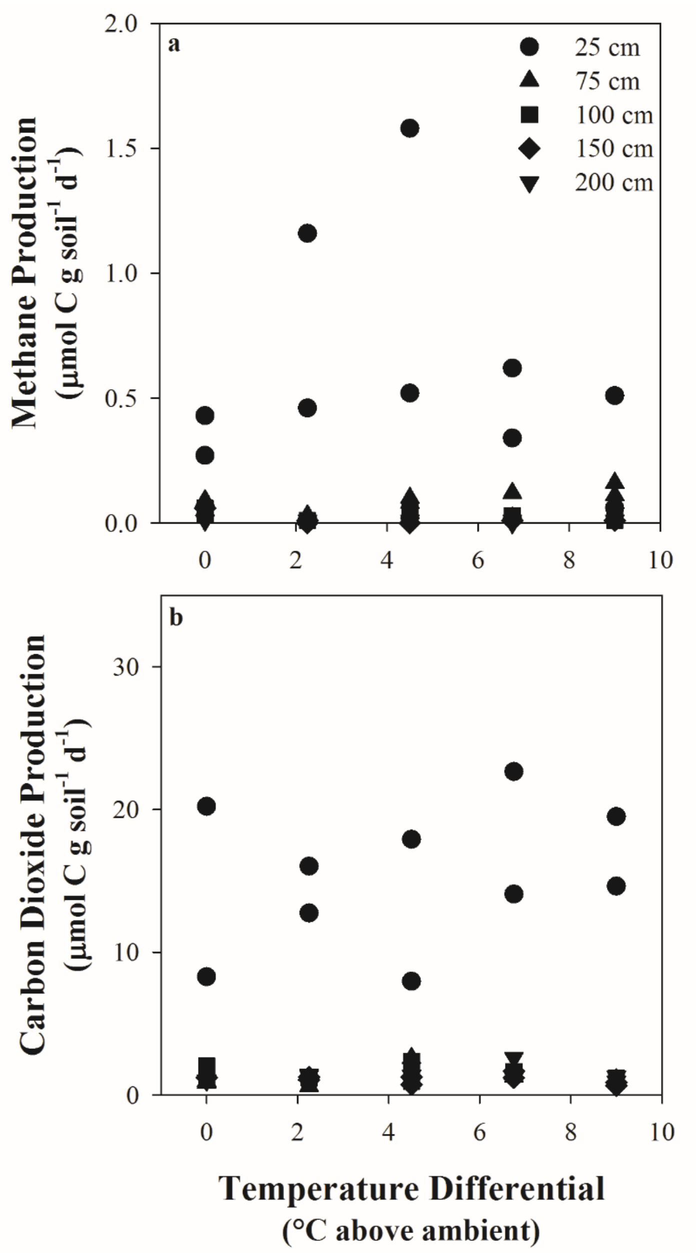

Supplementary Figure 3: CH_4 and CO_2 production in legacy effect incubations

Test for legacy effects of experimental warming on (a) CH_4 and (b) CO_2 production from peat samples taken at multiple depths and anaerobically incubated at a common temperature (20°C) after approximately 13 months of DPH. The circles indicate results from peat collected from 25 cm, triangles indicate results from 75 cm, squares indicate results from 100 cm, diamonds indicate results from 150 cm, and inverted triangles represent results from 200 cm. Data are plotted against the temperature treatment from which the peat was collected. Note the lack of significant temperature response across all depths.

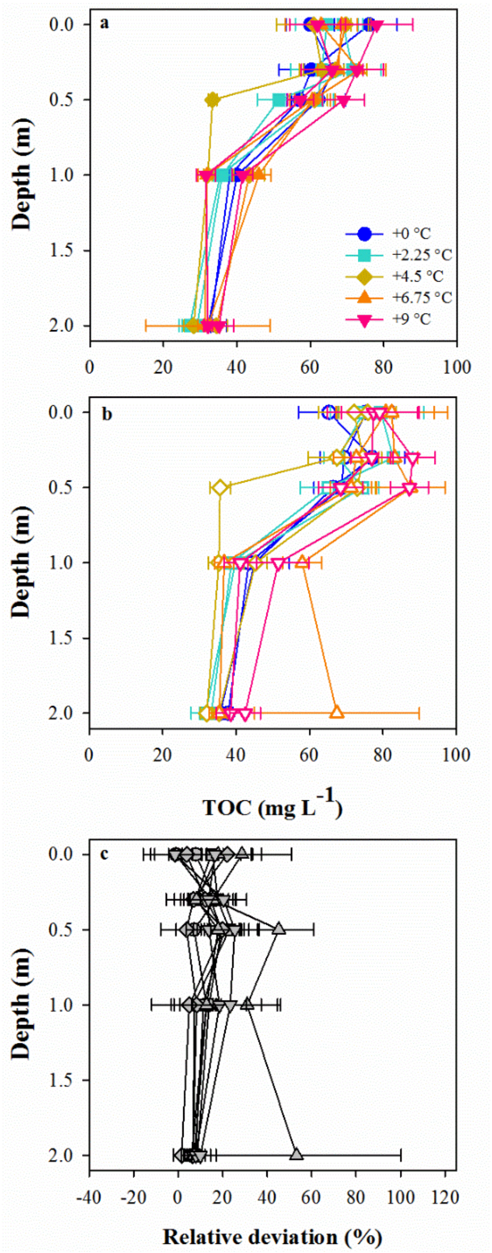

Supplementary Figure 4: Total dissolved organic carbon (DOC) concentrations

DOC concentrations in the peat porewater (a) prior to and (b) during deep peat heating. The relative deviation of DOC (c) was calculated to account for pre-treatment differences in DOC concentrations across plots. The deviation was calculated by dividing the DOC concentration at a given temperature treatment and a given depth by the mean DOC concentration at that same depth in the two control (0° C) plots prior to DPH (c). Points represent the averages of weekly (pre-DPH) or biweekly (during DPH) sampling and standard deviations of samples from all time points are indicated by the error bars. The blue circles represent results from the control (+0°C) plot, turquoise squares represent results from the +2.25°C treatment, gold diamonds represent results from the +4.5°C treatment, orange triangles represent results from the +6.75°C treatment, and magenta inverted triangles represent results from the +9°C treatment.

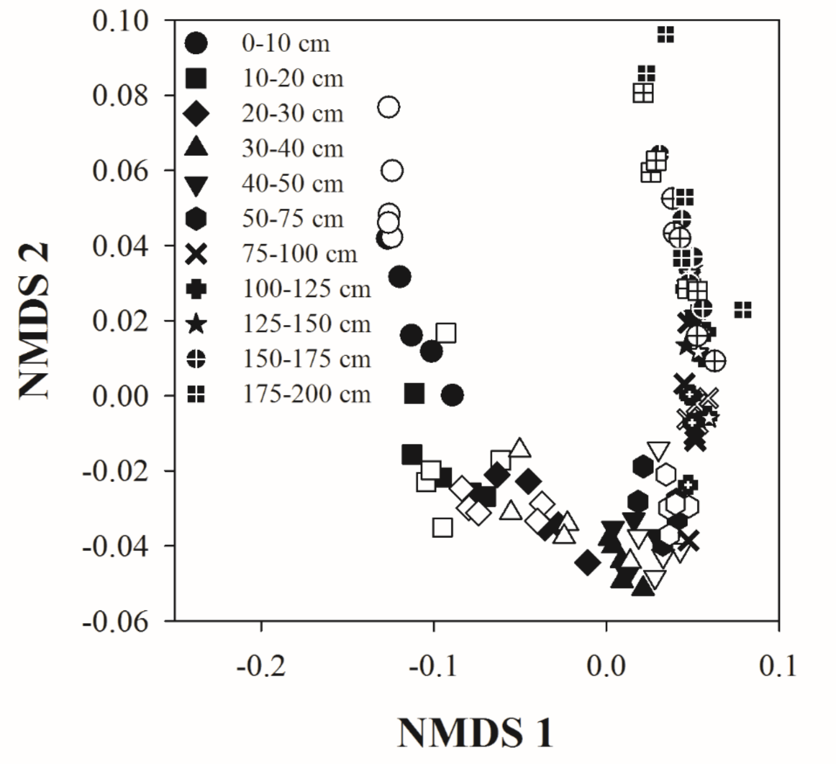

Supplementary Figure 5: Microbial community structure

Depth dependence of soil microbial community structure in SPRUCE site enclosures prior to (closed symbols) and after (open symbols) exposure to deep peat heating (DPH). Circles represent 0-10 cm, squares represent 10-20 cm, diamonds represent 20-30 cm, triangles represent 30-40 cm, inverted triangles represent 40-50 cm, hexagons represent 50-75 cm, x's represent 75-100 cm, **+** represent 100-125 cm, stars represent 125-150 cm, crossed circles represent 150-175 cm, and crossed squares represent 175-200 cm. Community structure exhibits strong vertical stratification in the peat column as visualized in a nonparametric multidimensional scaling plot (NMDS). Pairwise community distances were determined using the weighted Unifrac algorithm. A total of 5.35 million of rRNA gene sequences were normalized by cumulative sum scaling (CSS) methods and grouped by depth. As shown in Figure 4, no significant effect of temperature treatment or time is observed on community diversity or composition.

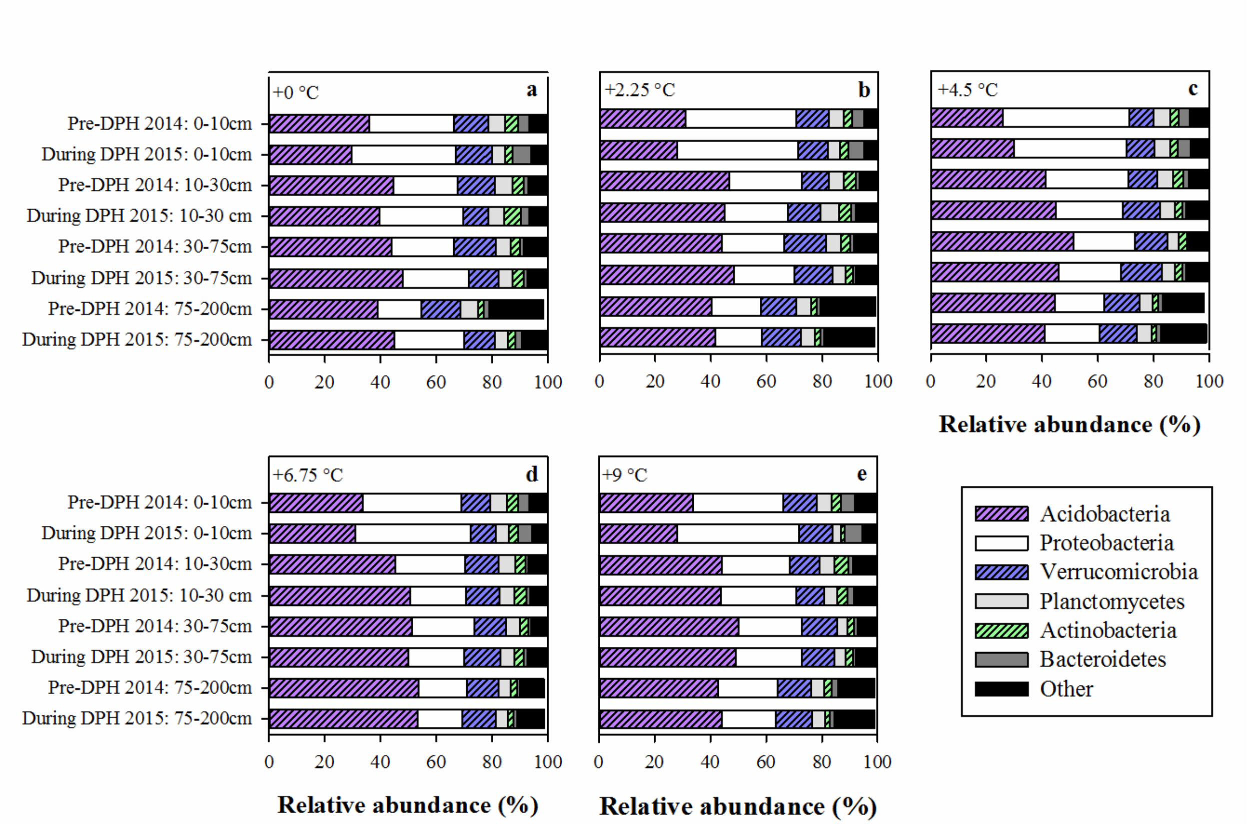

Supplementary Figure 6: Phylum level microbial relative abundances

Depth dependence of soil microbial groups detected at the phylum level (> 1 % divergence in gene sequences) in (a) control, (b) +2.25°C, (c) +4.5°C, (d) +6.75°C, and (e) +9°C plots prior to (2014) and after (2015) exposure to deep peat heating (DPH). Bars are stacked by date such that pre-DPH (2014) and during DPH (2015) results are proximate. Purple cross hatching represents results for *Acidobacteria*, solid white bars represent results for *Proteobacteria*, blue cross hatching represents results for *Verrucomicrobia*, solid light gray represents results for *Planctimycetes*, green cross hatching represents results for *Actinobacteria*, dark gray solid represents results for *Bacteroidetes*, and solid black represents results for all other phyla. The majority of microbial populations (~70%) are taxonomically affiliated to *Proteobacteria* and *Acidobacteria* phyla. A total of 5.35 million of rRNA gene sequences were assigned to the greengenes database by RDP Classifier at 50% confidence thresholds. Phyla which represented < 1% of relative abundance were not displayed and are summarized as Other.

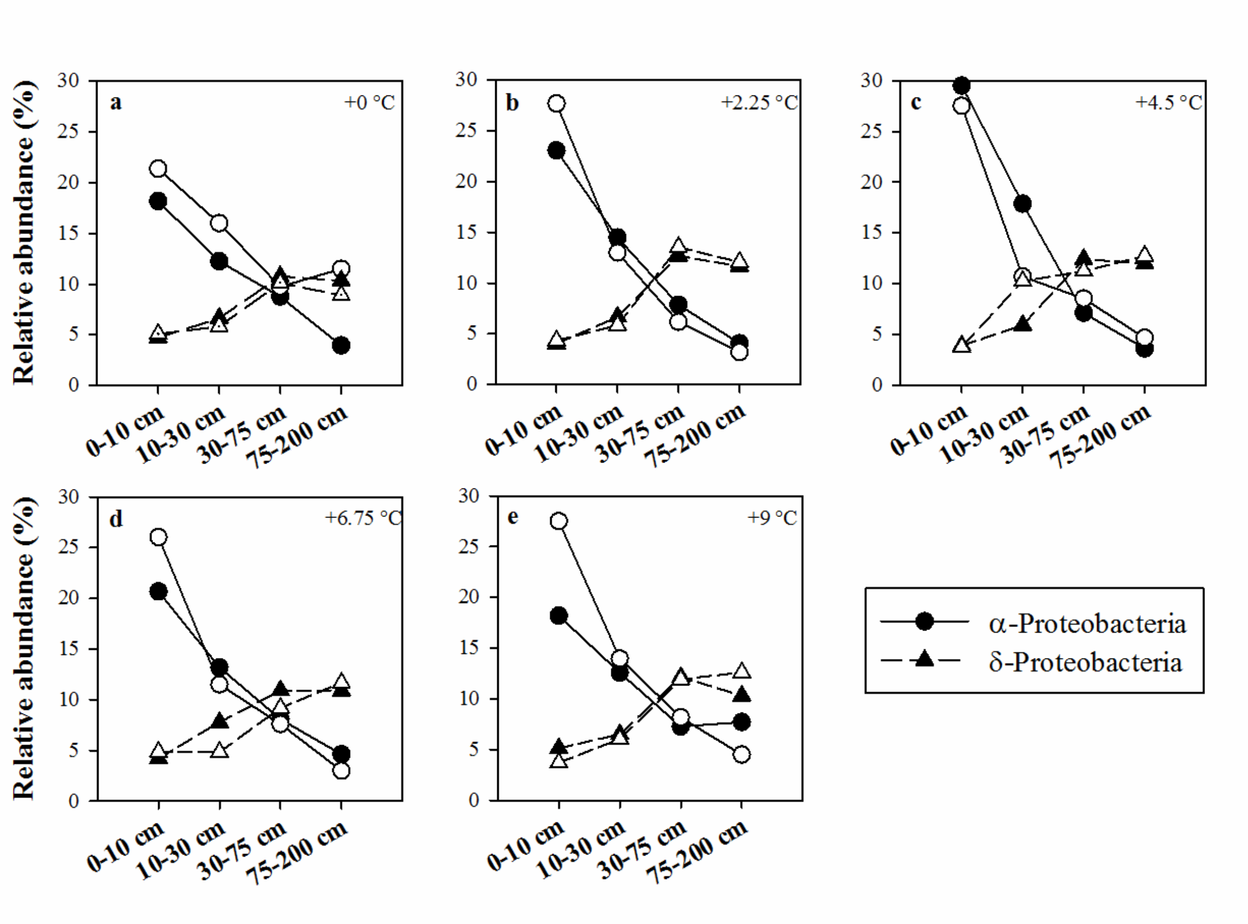

Supplementary Figure 7: *Alphaproteobacteria* and *Deltaproteobacteria* abundances

Depth dependence of soil microbial groups detected at the class level in (a) control, (b) +2.25°C, (c) +4.5°C, (d) +6.75°C, and (e) +9°C plots prior to (closed symbols) and after (open symbols) exposure to deep peat heating (DPH). Putative aerobic heterotrophs affiliated with the *Alphaproteobacteria* (circles) decreased in average relative abundance with depth, while putative anaerobes in the *Deltaproteobacteria* (triangles) increase with depth. Solid lines connect symbols for *Alphaproteobacteria* and dashed lines connect symbols for *Deltaproteobacteria*.

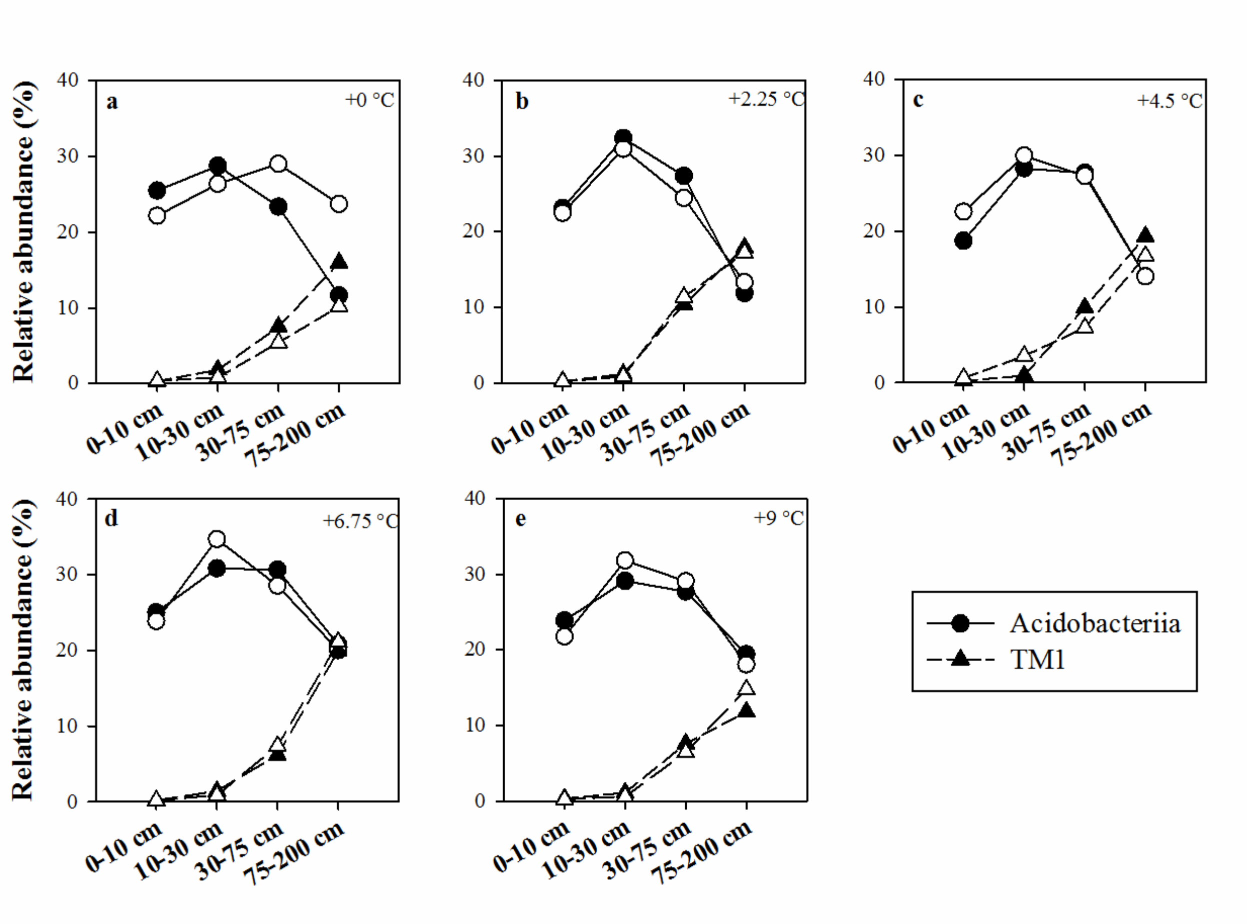

Supplementary Figure 8: Comparison of *Acidobacteriia* and TM1 relative abundances

Depth dependence of soil microbial groups affiliated with Class Acidobacteria in (a) control, (b) +2.25°C, (c) +4.5°C, (d) +6.75°C, and (e) +9°C plots prior to (closed symbols) and after (open symbols) exposure to deep peat heating (DPH). Circles denote *Acidobacteriia* and triangles denote TM1. Putative aerobic heterotrophs affiliated with the *Acidobacteriia* decreased in relative abundance with depth, while putative anaerobes in the TM1 class increase with depth. Solid lines connect symbols for *Acidobacteriia* and dashed lines connect symbols for TM1.

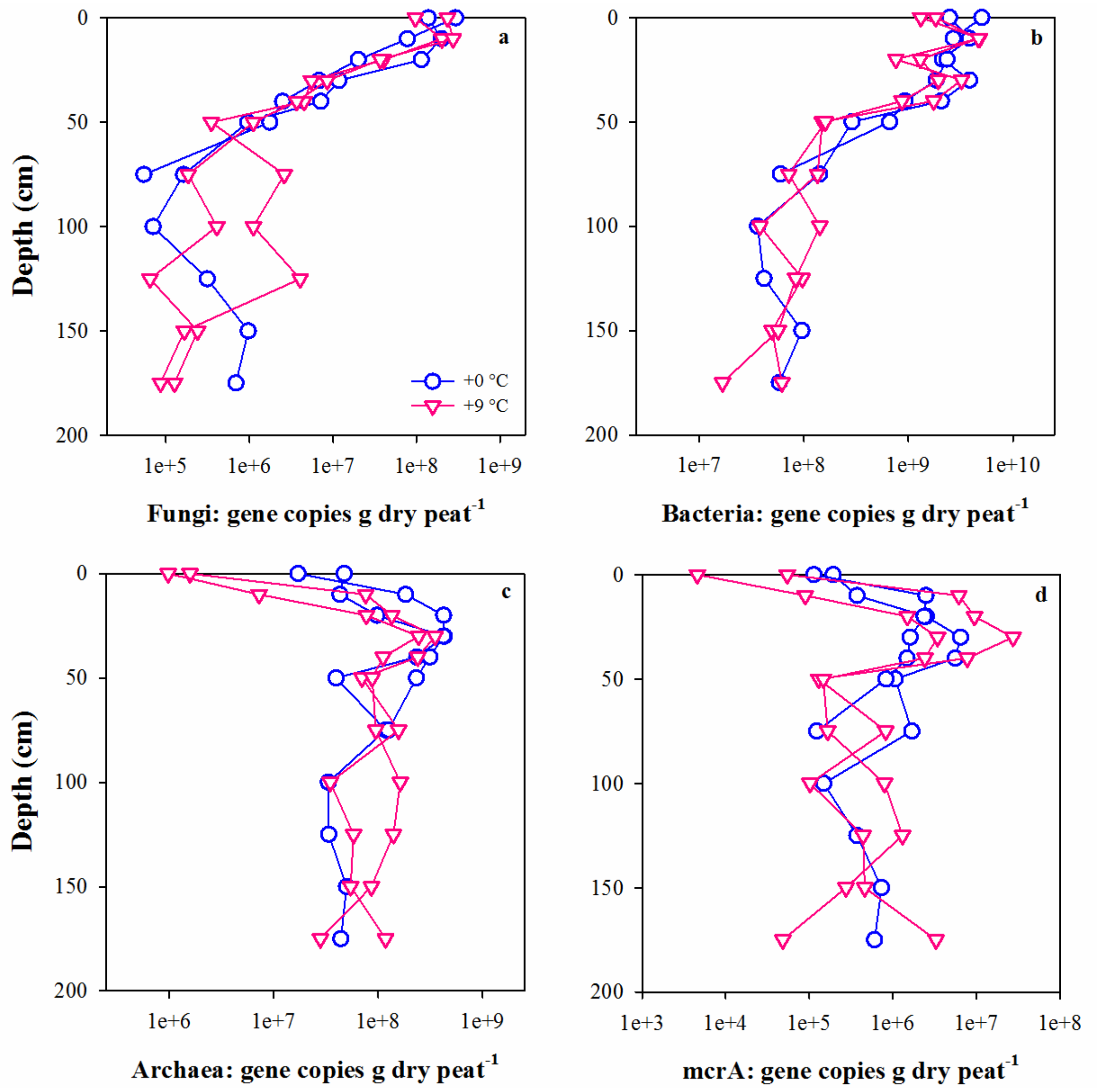

Supplementary Figure 9: Abundances of microbial groups in treatment plots

The abundance of (a) fungal, (b) bacterial, (c) archaeal and (d) *mcrA* gene copies were determined by quantitative PCR using primers targeted to amplify their respective SSU rRNA genes, and targeting the *mcrA* gene for methanogen populations. Microbial abundance is expressed for core samples from control (+0^0C) and +9^0C plots as gene copies per gram dry peat. Magenta circles represent results from control temperature treatment (0°C) and blue inverted triangles represents results from +9°C treatment plots. After thirteen months of deep peat heating (DPH) treatment, the *in situ* abundance of microbial groups (bacteria, archaea, fungi, and methanogen populations) shows no clear response to temperature, while strong vertical stratification is observed with peat depth.

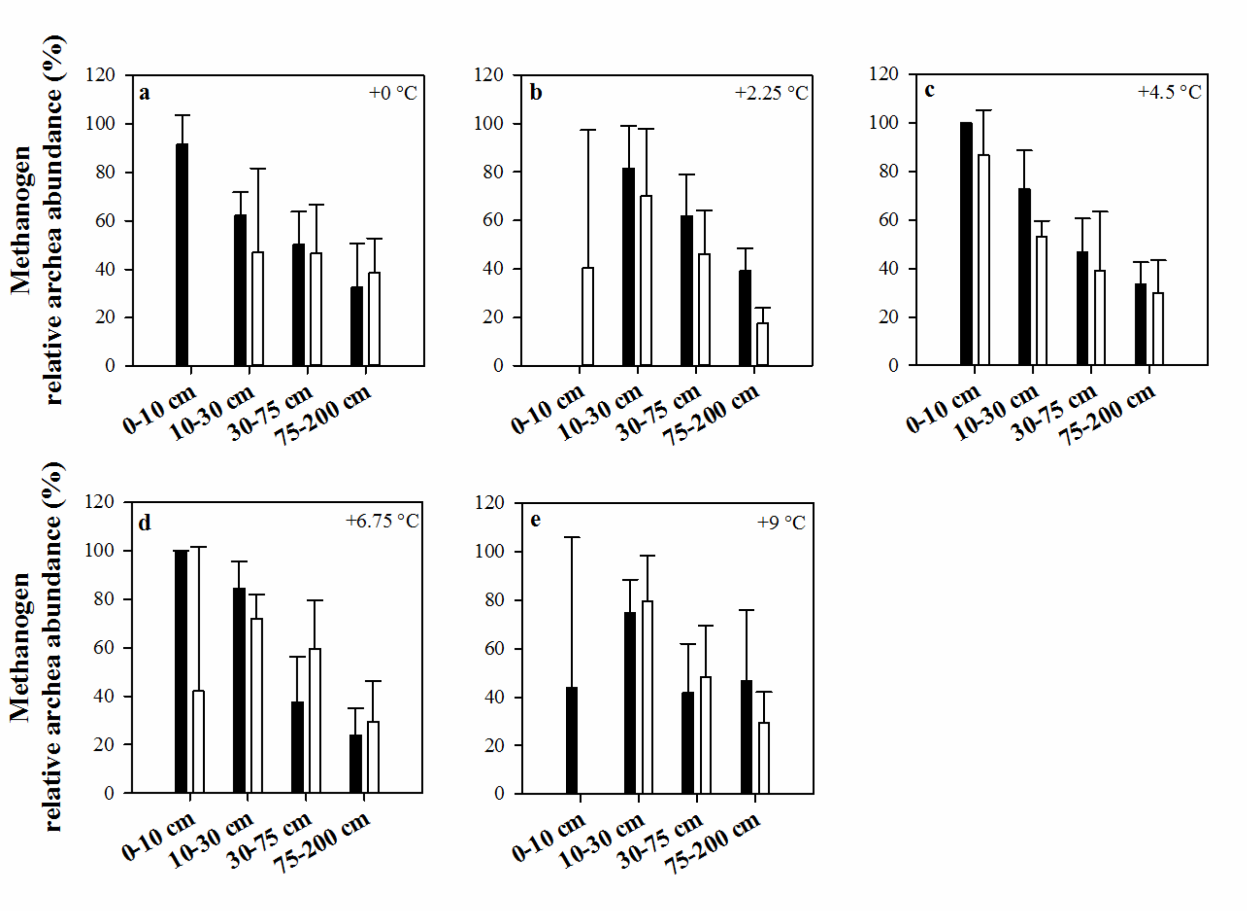

Supplementary Figure 10: Relative abundances of methanogens out of total Archaea

Depth dependence of known methanogenic Archaeal groups in treatment plots prior to and after exposure to deep peat heating (DPH) in (a) control (+0°C), (b) +2.25°C, (c) +4.5°C, (d) +6.75°C, and (e) +9°C plots. Apparent zero abundances (*e.g.* pre-DPH 0-10cm in the +2.25°C plot) reflect missing data. Abundance of known methanogens gradually decreases with peat depth, while no significant effect of temperature treatment or time on relative abundance of methanogens was observed. Pre-DPH is represented by closed bars and during DPH by open bars. Whiskers represent one standard deviation of replicate sample values.

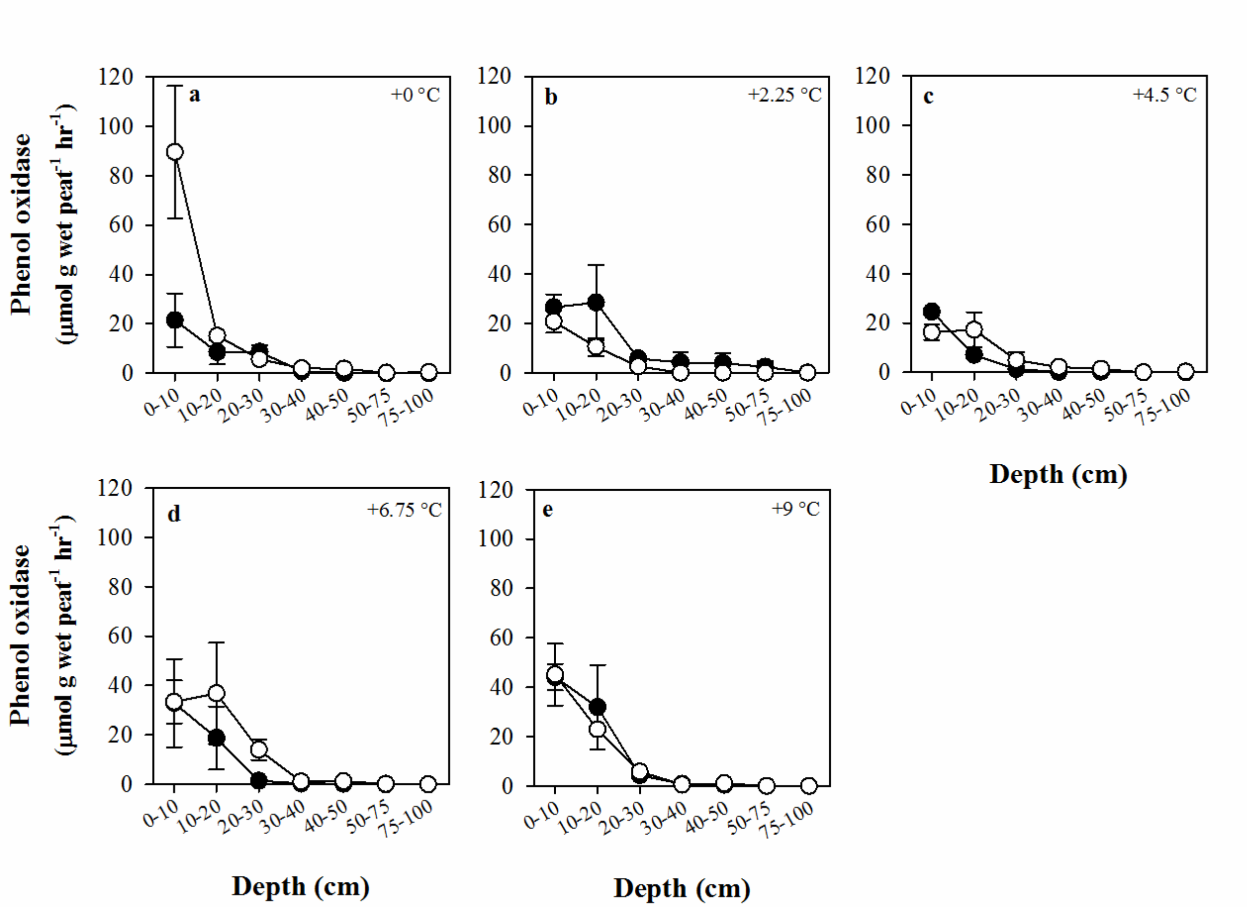

Supplementary Figure 11: Phenol oxidase enzyme activity changes with depth

Potential oxidative enzyme activity (phenol oxidase) in (a) control, (b) +2.25°C, (c) +4.5°C, (d) +6.75°C, and (e) +9°C plots prior to (June 2014, closed circles) and after (June 2015, open circles) exposure to deep peat heating (DPH). Temperatures indicated on panels indicate *in situ* temperature treatments. No significant effect of temperature or time on enzymatic activities was observed. Values are the mean of two cores with four technical replicates and error bars represent one standard deviation of these values.

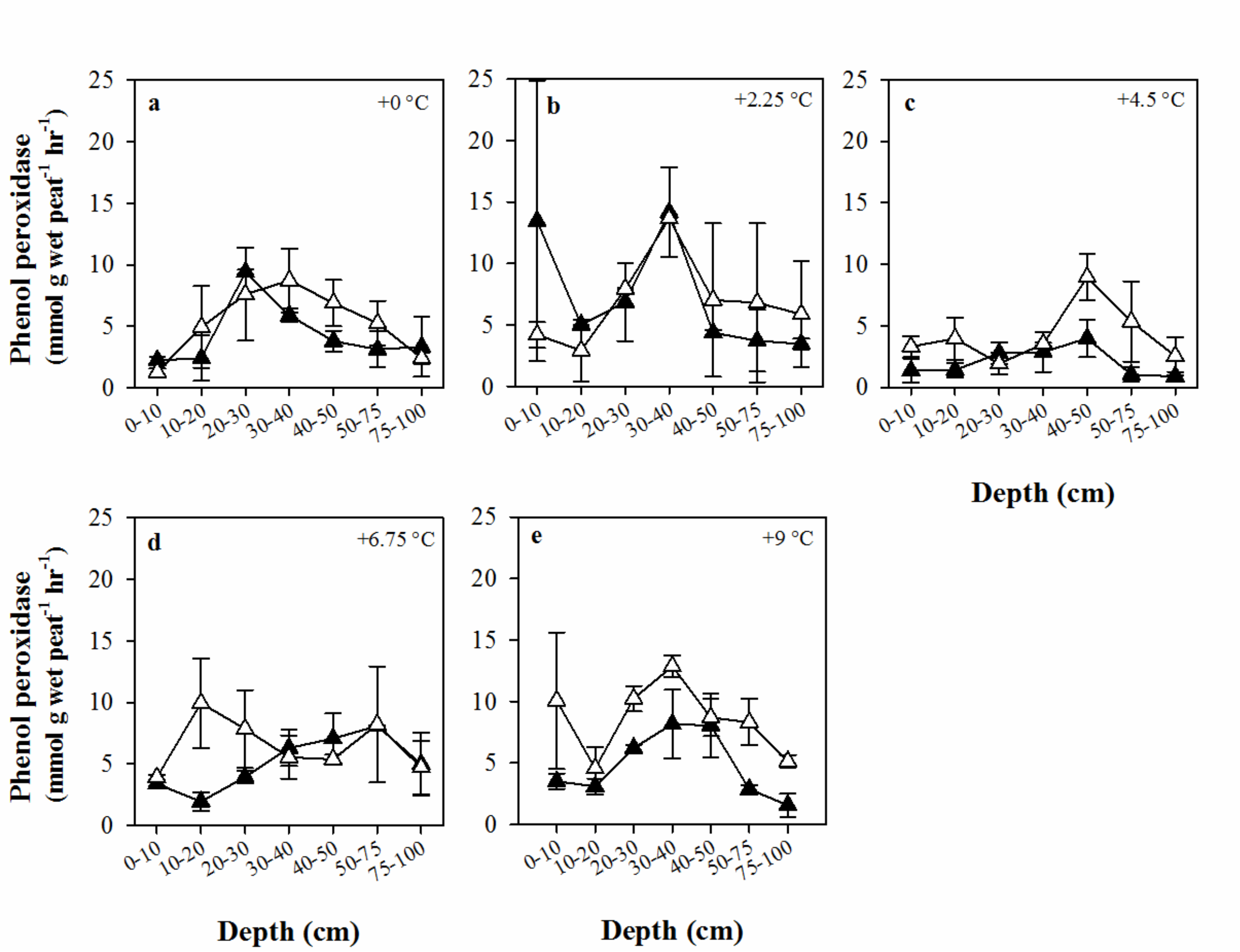

Supplementary Figure 12: Phenol peroxidase enzyme activity changes with depth

Potential oxidative enzyme activity (phenol peroxidase) in (a) control, (b) +2.25°C, (c) +4.5°C, (d) +6.75°C, and (e) +9°C plots prior to (June 2014, closed circles) and after (June 2015, open circles) exposure to deep peat heating (DPH). Temperatures indicated on panels indicate *in situ* temperature treatments. No significant effect of temperature or time on enzymatic activities was observed. Values are the mean of two cores with four technical replicates and error bars represent one standard deviation of these values.

Supplementary Table 1: Parameters for qPCR analysis of peat microbial communities

Microbial Group	Gene Target	Primer	Primer Reference	Organism for qPCR Standard
Eubacteria	16S	Eub 338<br>Eub518	Lane and Collins, 1991[1]<br>Muyzer et al., 1993[2]	*Escherichia coli*
Archaea	16S	915F<br>1059R	Yu et al., 2005[3]	*Methanococcus maripaludis* S2
Fungi	18S	nu-SSU-1196F<br>nu-SSU-1536R	Borneman & Hartin, 2000[4]	*Saccharomyces cerevisiae*
Methanogens	*mcrA*	mcrA_F<br>mcrA_R	Luton et al., 2002[5]	*Methanococcus maripaludis* S2

Supplementary References

1. Lane, D.J. & Collins, M.M. Current methods for detection of DNA/Ribosomal RNA hybrids. In A. Vaheri, R.C. Tilton, and A. Balow's (eds) *Rapid Methods and Automation in Microbiology and Immunology*, Springer-Verlag Berlin Heidelberg Germany, pp 54-75. (1991).
2. Muyzer, G., de Waal, E.C., & Uitterlinden, A.G. Profiling of complex microbial populations by denaturing gradient gel electrophoresis analysis of polymerase chain reaction-amplified genes coding for 16S rRNA. *Applied and Environmental Microbiology* **59,** 695-700 (1993).
3. Yu, Y., Lee, C., Kim, J., and Hwang, S. Group-specific primer and probe sets to detect methanogenic communities using quantitative real-time polymerase chain reaction. *Biotechnology and Bioengineering* **89,** 670-679 (2005).
4. Borneman, J. & Hartin, R.J. PCR primers that amplify fungal rRNA genes from environmental samples. *Applied and Environmental Microbiology* **66,** 4356-4360 (2000).
5. Luton, P.E., Wayne, J.M., Sharp, R.J., & Riley, P.W. the *mcrA* gene as an alternative to 16S rRNA in the phylogenetic analysis of methanogen populations in landfill. *Microbiology* **148,** 3521-3530 (2002).
